# Supersensitivity Psychosis and Its Response to Asenapine in a Patient with Delusional Disorder

**DOI:** 10.1155/2014/215732

**Published:** 2014-11-13

**Authors:** Ravi Philip Rajkumar

**Affiliations:** Department of Psychiatry, Jawaharlal Institute of Postgraduate Medical Education and Research (JIPMER), Pondicherry 605 006, India

## Abstract

Supersensitivity psychosis is a recognized complication of long-term antipsychotic treatment, in which patients develop new or reemergent psychotic symptoms, generally accompanied by dyskinetic movements, due to prolonged dopamine receptor blockade and resultant supersensitivity. Though it is most closely associated with schizophrenia and the use of typical antipsychotic agents, it has also been documented in patients with other diagnoses, and in those receiving atypical antipsychotics. There is no established treatment for this condition. In this paper, we describe a patient with persistent delusional disorder, jealous type, who developed a supersensitivity psychosis characterized by persecutory delusions, auditory hallucinations, and thought insertion in association with mild tardive dyskinesia. These symptoms resolved completely following six weeks of treatment with the second-generation antipsychotic asenapine, 20 mg/day. The mechanisms and implications of the patient's symptomatology and response are discussed.

## 1. Introduction

The term “supersensitivity psychosis” was first introduced into the psychiatric literature by Chouinard and Jones to refer to the emergence of psychotic symptoms in patients on long-term antipsychotic therapy [1, 2]. Such symptoms are thought to result from dopamine receptor supersensitivity consequent to chronic blockade of D_2_ dopamine receptors in the mesolimbic pathway, a mechanism analogous to that of tardive dyskinesia [1], though other processes such as cholinergic neuronal loss have been proposed [3]. Supersensitivity psychoses have been most commonly reported in patients with schizophrenia [1, 2, 4–7], though documented cases have also been reported in schizoaffective disorder [8], in bipolar disorder [9, 10], in intellectual disability [11], and even in subjects with no past history of mental illness [12]. Though its existence has been disputed by some authors [13], there has been a renewed interest in this condition with the realization that even newer “atypical” antipsychotics may be implicated in its causation [8, 14]. Criteria for its diagnosis have been developed by Fallon et al. [6, 15], based on both Chouinard's descriptions and further research. These include that though there is no approved treatment for supersensitivity psychosis, various approaches have been suggested, including risperidone [7, 16], clozapine [16], and anticonvulsants [17]. We report the case of a woman with delusional disorder who developed supersensitivity psychosis following ziprasidone therapy and who remitted when treated with the atypical antipsychotic asenapine.

## 2. Case Report

Mrs. D, a married woman aged 44, first presented to our clinic three years ago with seven years' continuous illness characterized by a single, well-systematized delusion of infidelity. This belief would lead to frequent quarrels with her husband, which sometimes escalated to physical violence. At presentation, she had never received any prior antipsychotic therapy, though she had received intermittent treatment with divalproex for “aggression.” A diagnostic evaluation revealed no medical or neurological causes of psychosis and no evidence of substance abuse or dependence. She was diagnosed to have persistent delusional disorder, jealous type (ICD code F22.0). As she was distressed about weight gain while on divalproex, she was offered treatment with aripiprazole, 15 mg/day, along with supportive couples therapy. However, she showed an inadequate response to this drug even after two months of continuous treatment. Her medication was changed to ziprasidone, which was initiated at 80 mg/day, which led to some improvement in her delusional belief over the next ten months. Due to a slight worsening of her symptoms in the context of a family dispute, this was increased to 100 mg/day. Within two weeks of the dose increase, she developed perioral and lingual dyskinetic movements. Her Abnormal Involuntary Movement Scale score was 4. As the movements were slight and were not causing significant distress, she agreed to continue ziprasidone at the same dose. This resulted in good symptomatic improvement for a month, after which she developed fresh symptoms which were quite unlike her initial presentation. Delusions of infidelity were absent; instead, she reported persecutory delusions towards her brother-in-law, second person auditory hallucinations of a male voice with derogatory content, and thought insertion. There were no pervasive mood changes associated with these symptoms, though she was distressed by the experience of thought insertion. A provisional diagnosis of paranoid schizophrenia was made, but given the relationship of her symptoms to her orolingual dyskinesias and the late age of onset, this was revised to supersensitivity psychosis as per Fallon and Dursun's criteria.

Ziprasidone was tapered and stopped, and she was treated with asenapine, initiated at 10 mg/day and slowly increased to 20 mg/day over the next month. After 6 weeks of treatment with 20 mg/day of asenapine, she reported a complete resolution of her new psychotic symptoms and had no dyskinetic movements. Her former delusion of infidelity did not return. She has been followed up for 18 months thereafter and has remained in remission, even after asenapine was decreased to 15 mg/day due to a complaint of daytime sedation. Her relationship with her husband remains satisfactory, and she has been able to return to work as a high school teacher.

## 3. Discussion

Our patient fulfilled all the revised diagnostic criteria for a supersensitivity psychosis proposed by Fallon et al. [6, 15]. These criteria are as follows.

1. Emergence of psychotic symptoms—delusions, hallucinations, and thought alienation phenomena—when on treatment with antipsychotics other than clozapine or quetiapine for over 1 year.
2. Presence of abnormal involuntary movements.
3. Compliance with antipsychotic treatment.
4. Absence of any significant life events or stressors.
5. Absence of any organic brain injury or substance abuse, including alcohol.
6. Significant interference in social and occupational functioning due to these symptoms.

In our patient, the likely offending agent was ziprasidone. Ziprasidone-induced supersensitivity psychosis has been reported earlier in an adolescent, though the time course of symptoms differed from that seen in our case [14]. Certain aspects of our patient's phenomenology are strikingly similar to earlier reports; for example, both thought alienation [18] and persecutory delusions [1, 12] have been documented in earlier cases of supersensitivity psychosis. As many patients with delusional disorder receive long-term antipsychotics, it is possible that at least some cases of apparent diagnostic transition from delusional disorder to schizophrenia—a phenomenon reported in as many as 42% of patients in one study [19]—may actually represent supersensitivity psychoses.

The choice of asenapine as a treatment modality in this patient was based on the fact that, like ziprasidone and aripiprazole, it is associated with lower rates of weight gain. However, there are theoretical reasons to believe that this drug may be useful in supersensitivity psychoses* per se,* because asenapine is a potent antagonist of serotonin 5-HT2A receptors [20], and 5-HT2A blockade may reduce the D2 receptor supersensitivity responsible for these patients' symptoms [21].

In conclusion, this case suggests that supersensitivity psychoses can occur in patients with delusional disorder and should be considered in patients whose psychotic symptoms apparently change while receiving antipsychotic therapy. Antipsychotics with strong 5-HT2A antagonism, such as asenapine, may be a safe and effective treatment option in such patients.

## References

[1] Chouinard G., Jones B. D. (1980). Neuroleptic-induced supersensitivity psychosis: clinical and pharmacologic characteristics. *The American Journal of Psychiatry*.

[2] Chouinard G. (1991). Severe cases of neuroleptic-induced supersensitivity psychosis. Diagnostic criteris for the disorder and its treatment. *Schizophrenia Research*.

[3] Miller R., Chouinard G. (1993). Loss of striatal cholinergic neurons as a basis for tardive and L-dopa-induced dyskinesias, neuroleptic-induced supersensitivity psychosis and refractory schizophrenia. *Biological Psychiatry*.

[4] Weinberger D. R., Bigelow L. B., Klein S. T., Wyatt R. J. (1981). Drug withdrawal in chronic schizophrenic patients: in search of neuroleptic-induced supersensitivity psychosis. *Journal of Clinical Psychopharmacology*.

[5] Hunt J. I., Singh H., Simpson G. M. (1988). Neuroleptic-induced supersensitivity psychosis: retrospective study of schizophrenic inpatients. *Journal of Clinical Psychiatry*.

[6] Fallon P., Dursun S. M. (2011). A naturalistic controlled study of relapsing schizophrenic patients with tardive dyskinesia and supersensitivity psychosis. *Journal of Psychopharmacology*.

[7] Kimura H., Kanahara N., Komatsu N., Ishige M., Muneoka K., Yoshimura M., Yamanaka H., Suzuki T., Komatsu H., Sasaki T., Hashimoto T., Hasegawa T., Shiina A., Ishikawa M., Sekine Y., Shiraishi T., Watanabe H., Shimizu E., Hashimoto K., Iyo M. (2014). A prospective comparative study of risperidone long-acting injectable for treatment-resistant schizophrenia with dopamine supersensitivity psychosis. *Schizophrenia Research*.

[8] Margolese H. C., Chouinard G., Beauclair L., Bélanger M.-C. (2002). Therapeutic tolerance and rebound psychosis during quetiapine maintenance monotherapy in patients with schizophrenia and schizoaffective disorder. *Journal of Clinical Psychopharmacology*.

[9] Steiner W., Laporta M., Chouinard G. (1990). Neuroleptic-induced supersensitivity psychosis in patients with bipolar affective disorder. *Acta Psychiatrica Scandinavica*.

[10] Witschy J. K., Malone G. L., Holden L. D. (1984). Psychosis after neuroleptic withdrawal in a manic-depressive patient. *American Journal of Psychiatry*.

[11] Sovner R. (1995). Thioridazine withdrawal-induced behavioral deterioration treated with clonidine: two case reports. *Mental Retardation*.

[12] Lu M.-L., Pan J.-J., Teng H.-W., Su K.-P., Shen W. W., Gonzàlez L., Demers D. (2002). Metoclopramide-induced supersensitivity psychosis. *Annals of Pharmacotherapy*.

[13] Singh H., Hunt J. I., Vitiello B., Simpson G. M. (1990). Neuroleptic withdrawal in patients meeting criteria for supersensitivity psychosis. *Journal of Clinical Psychiatry*.

[14] Jacob M. K., Ash P., Craighead W. E. (2012). Adolescent female with withdrawal psychosis following abrupt termination of ziprasidone. *European Child and Adolescent Psychiatry*.

[15] Fallon P., Dursun S., Deakin B. (2012). Drug-induced supersensitivity psychosis revisited: characteristics of relapse in treatment-compliant patients. *Therapeutic Advances in Psychopharmacology*.

[16] Chouinard G., Vainer J. L., Bélanger M.-C., Turnier L., Beaudry P., Roy J.-Y., Miller R. (1994). Risperidone and clozapine in the treatment of drug-resistant schizophrenia and neuroleptic-induced supersensitivity psychosis. *Progress in Neuropsychopharmacology and Biological Psychiatry*.

[17] Chouinard G., Sultan S. (1990). Treatment of supersensitivity psychosis with antiepileptic drugs: report of a series of 43 cases. *Psychopharmacology Bulletin*.

[18] Malcolm K. (1992). Supersensitivity psychosis with concurrent episodic vomiting. *British Journal of Psychiatry*.

[19] Whitty P., Clark M., McTigue O., Browne S., Kamali M., Larkin C., O'Callaghan E. (2005). Diagnostic stability four years after a first episode of psychosis. *Psychiatric Services*.

[20] Minassian A., Young J. W. (2010). Evaluation of the clinical efficacy of asenapine in schizophrenia. *Expert Opinion on Pharmacotherapy*.

[21] Bishop C., Tessmer J. L., Ullrich T., Rice K. C., Walker P. D. (2004). Serotonin 5-HT2A receptors underlie increased motor behaviors induced in dopamine-depleted rats by intrastriatal 5-HT2A/2C agonism. *Journal of Pharmacology and Experimental Therapeutics*.

